# Imidazopyridine-Based Thiazole Derivatives as Potential Antidiabetic Agents: Synthesis, In Vitro Bioactivity, and In Silico Molecular Modeling Approach

**DOI:** 10.3390/ph16091288

**Published:** 2023-09-13

**Authors:** Rafaqat Hussain, Wajid Rehman, Shoaib Khan, Aneela Maalik, Mohamed Hefnawy, Ashwag S. Alanazi, Yousaf Khan, Liaqat Rasheed

**Affiliations:** 1 Department of Chemistry, Hazara University, Mansehra 21120, Pakistan; rafaqathussain0347@gmail.com (R.H.); liaqatrasheed5142@gmail.com (L.R.); 2 Department of Chemistry, COMSATS University Islamabad, Islamabad 45550, Pakistan; aneela.maalik@comsats.edu.pk (A.M.); yousaf7n@gmail.com (Y.K.); 3 Department of Pharmaceutical Chemistry, College of Pharmacy, King Saud University, Riyadh 11451, Saudi Arabia; mhefnawy@ksu.edu.sa; 4 Department of Pharmaceutical Sciences, College of Pharmacy, Princess Nourah bint Abdulrahman University, P.O. Box 84428, Riyadh 11671, Saudi Arabia; asalanzi@pnu.edu.sa

**Keywords:** synthesis, thiazole, α-glucosidase, molecular docking

## Abstract

A new series of thiazole derivatives (**4a-p**) incorporating imidazopyridine moiety was synthesized and assessed for their in vitro potential α-glucosidase potency using acarbose as a reference drug. The obtained results suggested that compounds **4a** (docking score = −13.45), **4g** (docking score = −12.87), **4o** (docking score = −12.15), and **4p** (docking score = −11.25) remarkably showed superior activity against the targeted α-glucosidase enzyme, with IC_50_ values of 5.57 ± 3.45, 8.85 ± 2.18, 7.16 ± 1.40, and 10.48 ± 2.20, respectively. Upon further investigation of the binding mode of the interactions by the most active scaffolds with the α-glucosidase active sites, the docking analysis was accomplished in order to explore the active cavity of the α-glucosidase enzyme. The interpretation of the results showed clearly that scaffolds **4a** and **4o** emerged as the most potent α-glucosidase inhibitors, with promising excellent binding interactions with the active site of the α-glucosidase enzyme. Furthermore, utilizing a variety of spectroscopic methods, such as ^1^H-NMR, ^13^C-NMR, and HREI-MS, the precise structures of the synthesized scaffolds were determined.

## 1. Introduction

**Figure 1:**
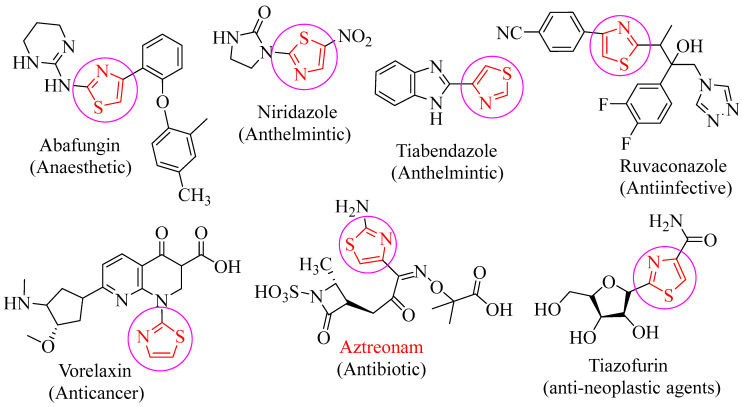
Thiazole skeleton-containing biologically active drugs.

Diabetes mellitus is characterized as a metabolic condition that has numerous non-infectious etiologies. It is described as persistent hyperglycemia and conflicts between the carbohydrate, protein, and fat metabolisms as a result of abnormalities in the secretion of insulin or its action, or both [1]. To avoid the consequences of diabetes, including peripheral arteriopathy, retinopathy, heart coronary disease, nephropathy, and neuropathy, blood glucose levels must be normalized, especially postprandial hyperglycemia [2]. In numerous modern and conventional medical systems around the world, a wide range of synthetic and natural compounds have been identified as potential sources of anti-diabetic special effects. Many of them have a reputation for being excellent diabetes treatments [3]. Many medications used to treat type-2 diabetes today not only have side effects but also have limited tolerability and efficacy and are not frequently successful in maintaining normal glucose levels in the blood [4,5]. The primary job of α-glucosidase (a member of the glycosyl hydrolases) is to hydrolyze the non-reducing terminal, α-D-glucosidase (1,4-linked), to facilitate the α-D-glucose discharge. The α-glucosidase enzyme has attracted particular attention among pharmaceutical and medicinal chemists, as it was found in earlier investigations that inhibiting its enzymatic potential delayed glucose absorption and decreased postprandial blood glucose levels. The inhibitors of α-glucosidase are crucial in preventing postprandial hyperglycemia and delaying or inhibiting the digestion and absorption of carbohydrates [6]. As a result, they have a wide range of applications, many of which may be helpful in type-2 diabetes treatment. Due to their distinct biological characteristics, heterocyclic molecules are of particular attention to chemists (medicinal). The most prevalent and essential analogs that exist exclusively in numerous pharmaceutical drugs, natural bioactive materials, and natural products are heterocyclic molecules that contain nitrogen [7]. The design and development of synthesis methods and novel technologies have been sparked by the discovery that N-heterocycles are a crucial structural entity in a number of physiologically potent compounds. Thiazole is a component of about 200 naturally occurring alkaloids that have been found thus far in various plants, microbes, and animals [8]. Among N-containing heterocyclic compounds, numerous substituted thiazoles have been produced in an effort to uncover more potential thiazole-based drugs [9]. The thiazole core skeleton-containing derivatives have been reported to show significant biological activities such as anti-diuretic, anti-Alzheimer, and anti-bacterial activities [10]. The hybrid analogs based on the thiazole moiety are also used as pharmacologically intriguing derivatives and have been shown to exhibit interesting biological effects, including those that have anti-inflammatory [11], anti-microbial [12,13], anti-hypoxic [14], analgesic [15,16], anti-cancer [17,18], anti-asthmatic [19,20], and anti-hypertensive activities [21]. Owing to the broad spectrum of the biological profile of the thiazole ring, it was also discovered that many commercially available biologically active medications, including ruvaconazole, abafungin, vorelaxin, tiabendazole, ruvaconazole, tiazofurin, aztreonam, and azereonam have the thiazole skeleton in their structures (Figure 1) [22,23].

**Figure 2:**
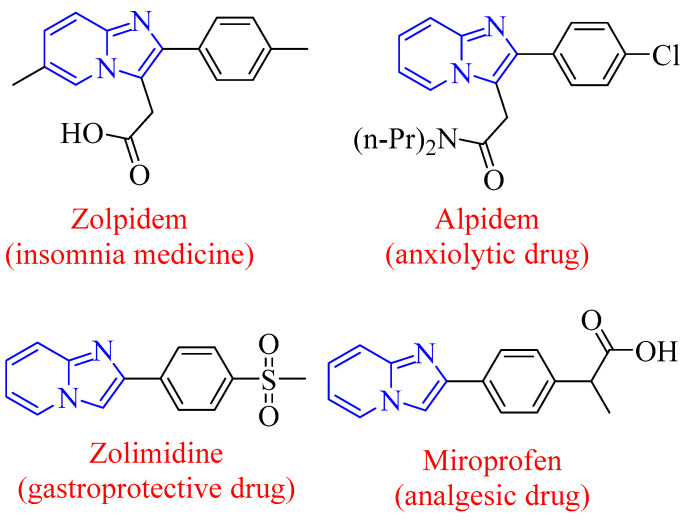
Medicinal significance of drugs containing the imidazopyridine moiety.

Additionally, essential for the study of their biological and pharmaceutical applications, the N-containing heterocyclic compounds have gained much attention [24]. One in particular is imidazopyridine, with rings of both imidazole and pyridine, which constitutes a typical, preferred scaffold, containing antifungal properties and working as an antagonist to the H1 receptor as well as a kinase and anti-lipase inhibitors (Figure 2) [25].

**Figure 3:**
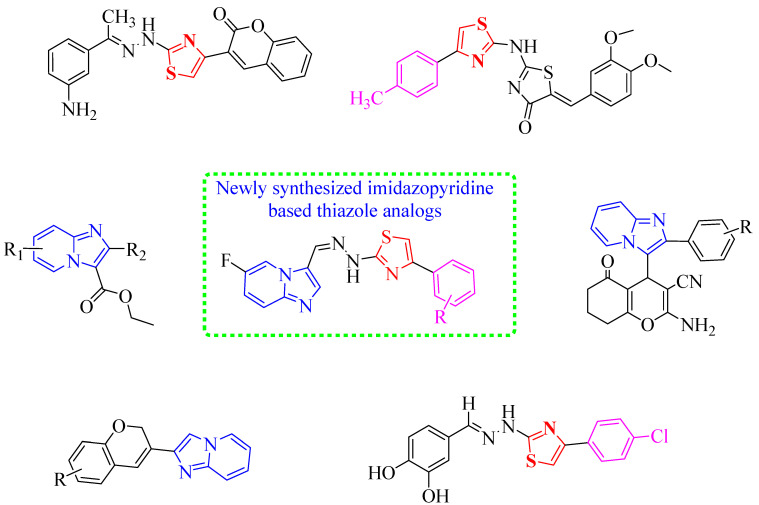
Rationale of the current work.

In order to design and synthesize hybrid analogs as potential anti-diabetic agents, the molecular hybridization technique has been widely used. This method mainly involves combining two or more distinct pharmacophore components into a single molecule with the same backbone. These hybrid molecules could be better than standard drugs. The physiologically significant heterocyclic moieties, including imidazopyridine bearing thiazole [26,27], and, as in this study, both thiazole [28,29,30] and imidazopyridine [31,32,33], are currently being combined through molecular hybridization to create new hybrid compounds (Figure 3).As was previously mentioned, the imidazopyridine and thiazole moieties are crucial as potential anti-diabetic agents, so hybrid analogs (**4a-4p**) with combinations of these two moieties were afforded and then tested for their ability to inhibit alpha-glucosidase in vitro (Figure 3).

## 2. Results

### 2.1. Chemistry

**Scheme 1:**
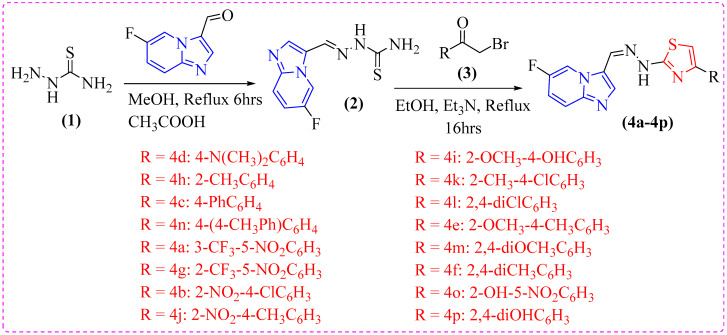
Synthesis of imidazopyridine-derived thiazole analogs.

In order to synthesize hybrid analogs of imidazopyridine-based thiazole (**4a-p**), thiosemicarbazide (**1**) was initially reacted to fluoro-containing imidazopyridine 3-carbaldehyde in methanol through the addition of a few drops of CH_3_COOH. The resulting mixture was refluxed for 6 hrs over a pre-heated sand bath to afford imidazopyridine-based thiosemicarbazone substrate (**2**) and further under wentcyclization on stirring with variably 2-bromoacetophenone (**3**) in Et_3_N and EtOH. The mixture of reaction was stirred and refluxed until it yielded the final product (TLC, 16 hrs reflux). The solvent was evaporated by employing low pressure to generate a solid residue of targeted analogs and then washed with n-hexane to form imidazopyridine-based thiazole analogs (**4a-p**) in a good yield (Scheme 1).

### 2.2. Biological Analysis (**4a-p**)

#### α-Glucosidase Inhibitory Activity

The inhibition profile of each synthesized scaffold was shown in vitro. The results were compared with acarbose as a reference. The inhibitory potential of the newly synthesized scaffolds ranged from 5.57 ± 3.45 to 63.46 ± 5.28 μM in comparison to the acarbose reference (IC_50_ = 48.71 ± 2.65 μM).

Generally, it was summarized based on the aforementioned observation that theα-glucosidase activity of imidazopyridine-based thiazoles (**4a-p**) was largely affected by the introduction of attached substituents; the nature and location with respect to the phenyl ring can vary. Moreover, the inhibitory potential of synthetic imidazopyridine-based thiazole analogs was also impacted by the change in the number of attached substituent(s) around the aryl parts (Table 1).

### 2.3. Molecular Docking Study

**Figure 4:**
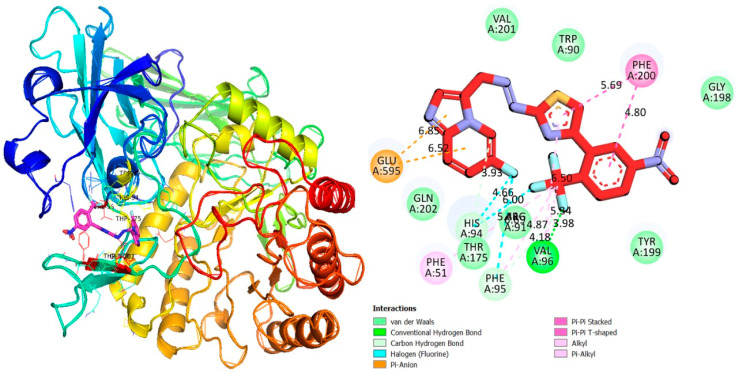
The protein–ligand interaction profile of the most potent scaffold **4g** against the targeted α-glucosidase enzyme and its 3D and 2D diagram.

**Figure 5:**
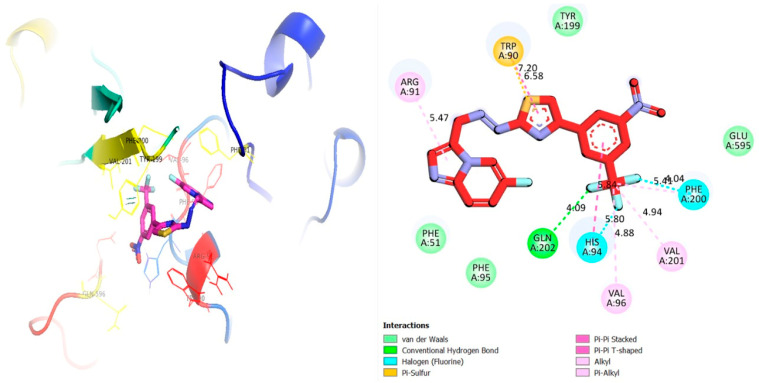
The PLI of the second-most active analog **4a** against the targeted α-glucosidase enzyme and its 3D and 2D diagram.

**Figure 6:**
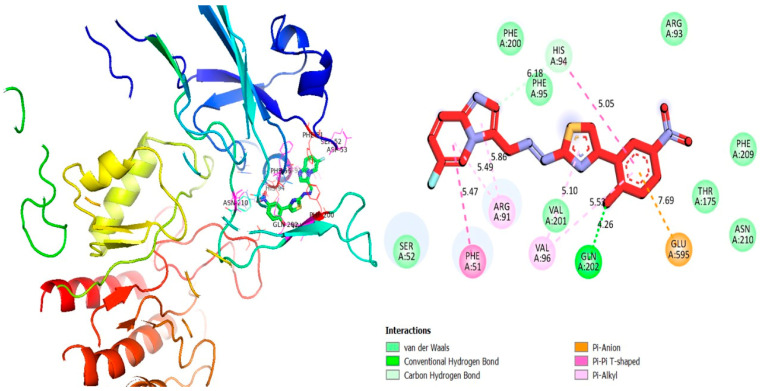
The PLI of the third-most active analog **4o** against the targeted α-glucosidase enzyme and its 3D and 2D diagram.

**Figure 7:**
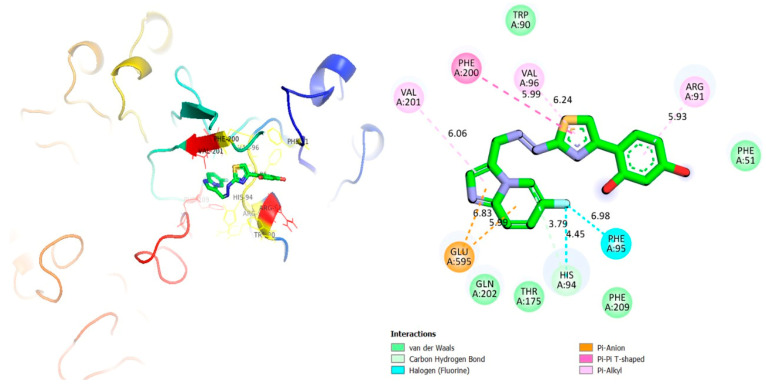
The PLI of thefourth-mostactive analog **4p** against the targeted α-glucosidase enzyme and its 3D and 2D diagram.

A molecular docking study has been performed to assess the correlation between in vitro and in silico studies of all the active synthesized **4g**, **4a**, **4o**, and **4p** analogs. All of these active compounds subsequently bind effectively in the target’s active site with a variety of binding affinities and also correlate with in vitro studies. All of these active analogs have the same chemistry with slight variations in different positions. Different interactions with the target alpha-glucosidase active sites were shown by these various functional moieties at different positions of active analogs (Figure 4, Figure 5, Figure 6 and Figure 7).

## 3. Discussion

### 3.1. Structure–Activity Relationship (SAR) Study for α-Glucosidase Inhibitory Profile

Structure–activity relationship (SAR) studies were conducted for the newly synthesized analogs (**4a-p**) by bringing substituent(s) around the aryl part, and any variation in inhibitory potentials was due to variation in number, position, and ED/EW nature of the substituent(s) linked to the aryl part while keeping the imidazole and thiazole rings constant (Table 2).

**Figure 8:**
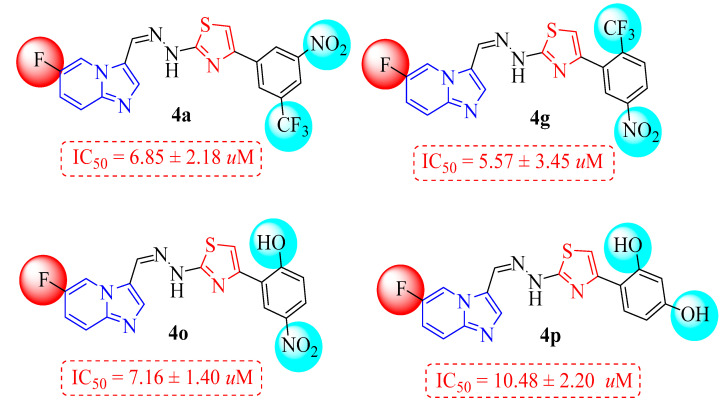
SAR studies of **4a**, **4g**, **4o**, and **4p** analogs.

The compounds (**4a**, **4g**, **4o**, and **4p**) bearing substituent(s) of either strong EWG nature, such as -CF_3_ and -NO_2_, or strong H-bond (-OH) making substituent(s) attached to variable positions of the extended aromaticbenzene ring were recognized asdemonstrating significant potentcy, when compared to the remaining analogs of the current synthesized series. The scaffold **4g** bearing *ortho*-CF_3_ and *meta*-nitro substituents at the phenyl ring was shown to exhibit many times more potency than the standard acarbose drug, as well as the rest of the analogs of the synthesized series. However, the analog **4a**, which also holds the same -CF_3_ and -NO_2_ moieties at the phenyl ring, has a different position of the -CF_3_ moiety around the aryl part and was found somewhat less potent than its counterpart **4g**, even through this scaffold **4a** was identified as the second-most potent inhibitor of the target among the series. The enhanced inhibitory potentials of both of these scaffolds, **4g** and **4a**, might be due to the attached substituents (-CF_3_ and -NO_2_) having a strong EW nature and are also capable of interacting through side-wise association with the target active sites. Further, the comparison of scaffolds **4g** (which holds 2-CF_3_ and 5-NO_2_ groups at the phenyl part) with analog **4o** (bearing 2-OH and 5-NO_2_ groups at the aryl part) demonstrated that the **4g** scaffold exhibited more enhanced inhibitory potentials than the **4o** analog. Both of these scaffolds differ from one another due to the nature of substituents (5g holds 2-CF_3_, while **4o** bears 2-OH group) at the 2-position of the phenyl ring. The better inhibitory potentials of scaffold **4g** revealed that the -CF_3_ moiety established better interactions than the -OH group with target active sites. Similarly, analog **4p** (which holds di-hydroxy at the 2,4-position of the phenyl ring) displayed many times more potency than the standard acarbose drug but was found less potent than its counterparts **4a**, **4g**, and **4o**. In addition, the comparison of analog **4o** (bearing 2-hydroxy and 5-NO_2_) with **4p** (bearing di-hydroxy at the 2,4-position) shows that analog **4o** exhibited enhanced inhibitory potentials owing to the attached EW -NO_2_ group at the 5-position, which interacts better with the target active sites (Figure 8).

**Figure 9:**
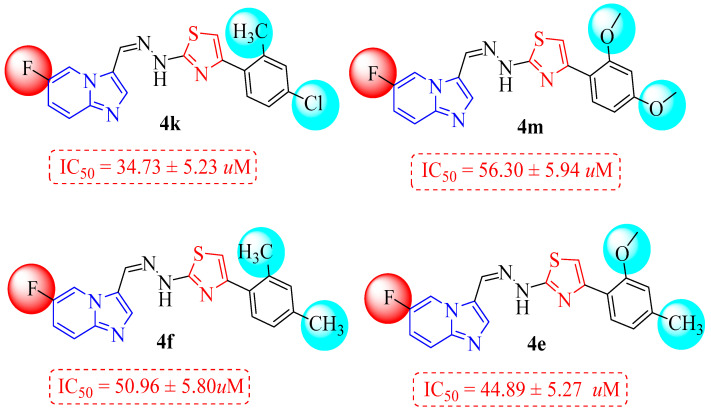
SAR studies of **4k**, **4m**, **4f**, and **4e** analogs.

It was revealed by the SAR studies that the attachment of substituent(s) (-CH_3_ and -OCH_3_,either alone or in combination) was capable of forming weak interactions with the target active sites and hence resulted in declined potency. Analog **4k**, which holds -CH_3_ at the *ortho*- and -Cl moieties at the *para*-position of the aryl part, showed good potency as compared to the standard drug. This better activity of analog **4k** was due to the strong e-withdrawing effects of the -Cl moiety, which makes the aryl ring more probed for interaction with target α-glucosidase active sites. Similarly, **4m** has lower potency than the standard drug, which might be due to the presence of two -OCH_3_ groups on the 2,4-position of the aryl part. These two methoxy groups have an electron-donating effect, and due to this, it will lower the inhibition activity. Moreover, if we compare analog **4m**, bearing di-OCH_3_ groups at the 2,4-position of the aryl part, with scaffold **4f**, bearing di-CH_3_ moieties at the 2,4-position of the aryl part, the analog **4f** displayed superior activity than that of analog **4m**. This difference was due to the fact that the methyl group is the ring activator group which is less electronegative than the oxygen of the methoxy group and a charge is not produced on the aryl ring as compared to the methoxy groups of **4m**; therefore, the inhibition activity of **4f** is somewhat better than **4m**. However, compound **4e** holds both the *ortho*-OCH_3_ and *para*-CH_3_ groups and makes a somewhat better competitor of α-glucosidase activity as compared to the standard drug (Figure 9).

**Figure 10:**
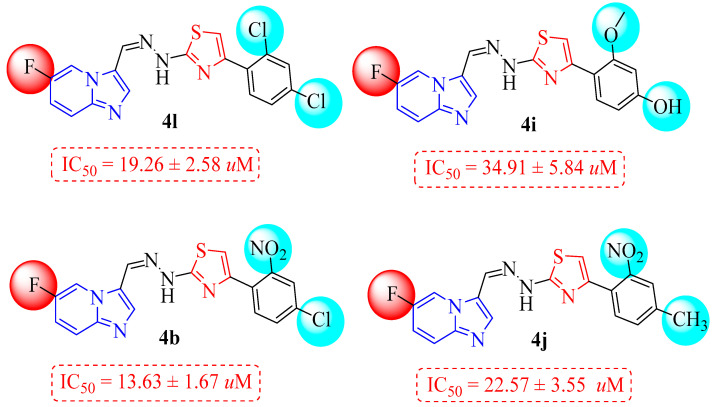
SAR studies of **4b**, **4i**, **4j**, and **4l** analogs.

Moreover, the inhibition profiles of the newly afforded scaffolds were found to be encouraging by attachment of -Cl, -NO_2_, and -OH groups, either alone or in combination, at variable positions of the aryl part. The analog **4b**, bearing the-NO_2_ moiety at the *ortho-* and the -Cl moieties at the *para*-position of the phenyl moiety has emerged as the potent inhibitor of the α-glucosidase enzyme (IC_50_ = 13.63 ± 1.67 μM) in comparison to the reference drug (48.71 ± 2.65). This elevation in activity of scaffold **4b** might be owing to the attached -NO_2_ and -Cl groups having strong e-withdrawing natures. Similarly, analog **4l**, bearing di-Cl groups at the 2,4-position of the aryl part, shows two-fold more potency as compared to the standard drug, while showing a slightly lower potency as compared to analog **4b**. This better potency was due to the attached -Cl group, which is a good e-withdrawing group that will enhance the potency of the molecule. Moreover, the compound **4j** has also shown two-fold more potency as compared to the standard drug due to the presence of an electron-withdrawing group of -NO_2_ at the 2-position of the phenyl ring. The inhibition profile of scaffold **4i** is also good/moderate with MIC value (34.91 ± 5.84) as compared to the standard acarbose (48.71 ± 2.65) drug, due to the presence of -OCH_3_ and -OH, because the -OCH_3_ group also increases the inhibitory potential to some extent, as well as the -OH holds the tendency of making an H-bond with the target active sites and therefore increases the potency of the molecule. This difference found in the inhibition profiles of both scaffolds **4b** and **4l** might be due to the stronger EW nature possessed by -NO_2_ than that of the -Cl moiety, which holds a weaker EW effect, as well as the fact that the-NO_2_ groups also have a tendency to established strong side-wise interactions by involving its oxygen with the target active sites and hence, enhanced the enzymatic potentials when the results are compared to the reference drug (Figure 10).

**Figure 11:**
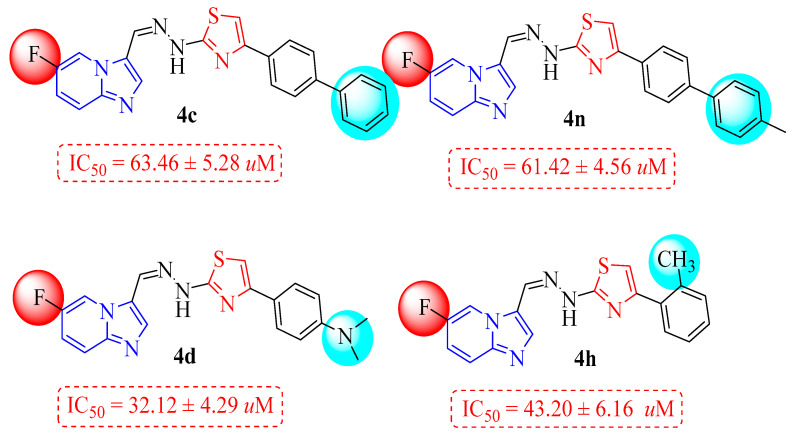
SAR studies of **4c**, **4d**, **4h**, and **4n** analogs.

Nonetheless, the SAR studies suggested that the incorporation of groups of a bulky nature, such as -Ph, -toluene, and -N(CH_3_)_2_ moieties, at the *para*-position of the aryl part, resulted in a decrease in inhibitory potentials against the targeted *alpha*-glucosidase enzyme. Analog **4c**, bearing para-phenyl moiety at the aryl part, and analog **4n**, having p-tolyl moiety at the aryl part, emerged as the least potent competitors of the *alpha*-glucosidase enzyme among the series. This decreased inhibitory potential of both analogs **4c** and **4n** might be due to the bulky nature of attached substituent(s). Apart from this, compounds **4d** (holds -N(CH_3_)_2_ at the *para*-position) and **4h** (holds -CH_3_ at the *ortho*-position of the aryl part) showed moderately good results when compared with the standard acarbose drug. Hence, **4c** and **4n** showed reduced potency comparable with the standard drug, while compounds **4d** and **4n** showed moderately good potency as compared with the acarbose drug (Figure 11).

### 3.2. Molecular Docking Study

#### 3.2.1. Discussion

Through in vitro analysis, compound **4g** was found to be the most active. The targeted active analog sites interacted with the most active analog. The most potent analog’s detailed interactions reveal that this scaffold provided several significant interactions with the active α-glucosidase target sites, including Phe200 (pi-pi T shaped), Glu595 (pi-anion), Val96 (CHB, pi-alkyl and pi-pi stacked), Phe51 (pi-alkyl), Thr175 (carbon hydrogen bond), His94 (halogen (fluorine)), Phe95 (halogen (fluorine) and pi-alkyl), and Arg91 (pi-alkyl) interactions.

The PLI of the second-most effective scaffold **4a** showed that, despite a structural similarity with the most active analog **4g**, the two analogs differ only in the location of the -CF_3_ moiety in analog **4a**; it is attached at the meta-position of the aryl ring, whereas in analog **4g**, it is attached at the *ortho*-position of the aryl part of the thiazole. The different positions of the functional moiety -CF_3_ around the aryl part may be the cause of the reduction in the enzymatic potential, which makes both the active **4g** and **4a** analogs interact differently. The detailed PLI of the second-most active scaffold **4a** showed that this analog adopted various noteworthy interactions with the target α-glucosidase active sites, including Trp90 (pi-sulfur and pi-pi T shaped), Arg91 (pi-pi stacked), Gln202 (CHB), His94 (halogen (fluorine) and pi-pi stacked), Val96 (pi-alkyl), Val201 (pi-alkyl), and Phe200 (halogen (fluorine) and pi-alkyl) interactions.

According to the docking study, substituents that form strong hydrogen bonds with the target active sites also enhance the inhibition profile; as a result, analog **4o** (bearing ortho-hydroxy and meta-nitro moieties at the aryl part) established various important interactions with the target α-glucosidase active sites, such as Phe51 (pi-pi T shaped), Arg91 (pi-pi stacked), Val96 (pi-pi stacked and pi-alkyl), Gln202 (CHB), Glu595 (pi-anion), Phe95 (carbon–hydrogen bond), and His95 (pi-pi T shaped) interactions.

As an additional active inhibitor of the α-glucosidase enzyme, the analog **4p** with di-hydroxy substituents at the 2,4-position of the aryl part of the thiazole ring showed various significant important interactions, such as Arg91 (pi-alkyl), Phe200 (pi-pi T shaped), Val96 (pi-alkyl), Val201 (pi-alkyl), Glu595 (pi-anion), His94 (halogen (fluorine) and carbon–hydrogen bond), and Phe95 (halogen (fluorine)) interactions.

#### 3.2.2. ADME Analysis

**Graph 1:**
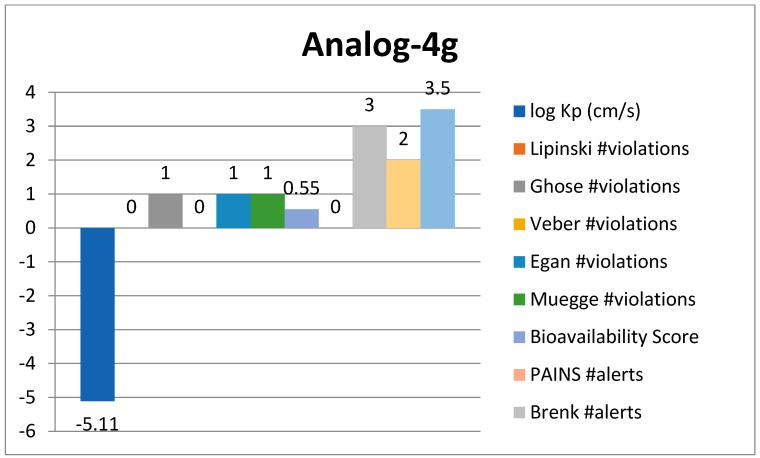
Representation of the drug-likeness properties of analog **4g**.

**Graph 2:**
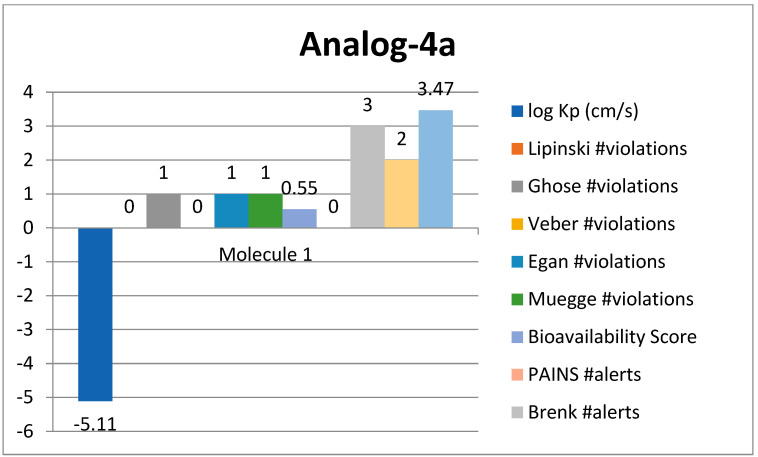
Representation of the drug-likeness properties of analog **4a**.

**Graph 3:**
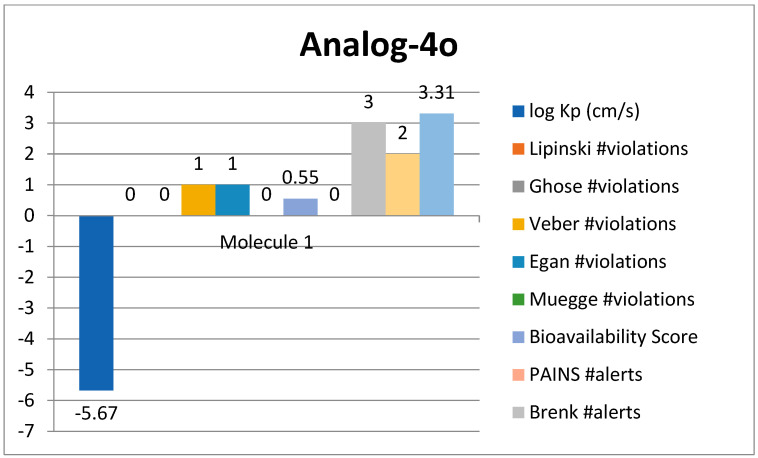
Representation of the drug-likeness properties of analog **4o**.

**Graph 4:**
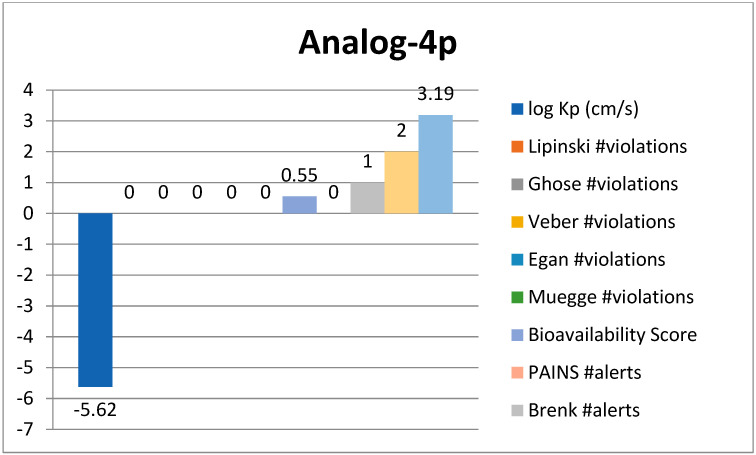
Representation of the drug-likeness properties of analog **4p**.

An ADME analysis was performed using the online software Swiss ADME in order to explore the better potentials of synthesized compounds having drug-likeness properties. The subjected analogs **4g**, **4a**, **4o**, and **4p** were identified by similar criteria, which showed varied drug-like properties as shown in Graph 1, Graph 2, Graph 3, Graph 4.

## 4. Materials and Methods

### 4.1. General Information

Using an electro-thermal melting point apparatus called the Stuart-SMP30 in open glass capillaries, the uncorrected melting points (°C) were ascertained. Nuclear magnetic resonance (NMR) spectra for 1H and 13C were obtained on a JEOL ECA-500 II NMR spectrometer at 600 MHz and 150 MHz, respectively. The chemical shifts represented in ppm downfield from the internal standard, tetramethylsilane, and the coupling constants (J) are given in Hz. Deuteriodimethyl sulfoxide was used as the solvent (DMSO-d6). The reactions were seen and the purity of the finished products was evaluated using thin-layer chromatography, utilizing silica-gel-precoated aluminum sheets (60 F254, Merck) and visualization with ultraviolet light (UV) at 365 and 254 nm.

### 4.2. General Procedure for Synthesis of Imidazopyridine-Based Thiazole Analogs (**4a-p**)

The synthesis of imidazopyridine-based thiazole was completed in two steps: In the first step, imidazopyridine-3-carbaldehyde (1 equivalent) was added to thiosemicarbazide (1) (1 equivalent) solution being stirred in methanol (10 mL) and CH3COOH (catalyst). The residue was refluxed for 6 hrs to deliver imidazopyridine-based thiosemicarbazone (2). In the last step, an intermediate (2) (1 equivalent) was subjected to cyclization with different substituted phenacyl bromide (3) (1 equivalent) in methanol (10 mL) and triethylamine (catalyst) to afford the targeted imidazopyridine-based thiazole analogs (**4a-p**).

### 4.3. Spectral Analysis (Provided in Supplementary Information)

#### 4.3.1. Assay Protocol for α-Glucosidase Inhibition

With slight modification to an earlier technique, the α-glucosidase inhibition profiles of the synthesized scaffolds were assessed spectrophotometrically [34]. Amounts of 10 μL of the sample, 120 μL of 100 mM potassium phosphate buffer, and 20 mL of a 0.5 U/mL α-glucosidase solution (0.3 mM, in buffer) were combined for each chemical. As an inhibitor, acarbose was commonly employed. Each combination was incubated at 37 °C for a separate period of 15 min. An amount of 20 μL of the substrate 4-nitrophenyl—D-α-glucopyranoside (pNPG) (5 mM) was added to the solution to start the enzymatic reaction. Once more, the mixture was incubated for 15 min at 37 °C. At that point, 80 μL of a 0.2 M sodium carbonate solution was administered to terminate the process. The mixture that included no enzymes also functioned as a blank sample. At 405 nm, the absorbance was finally calculated. To accurately measure the background absorbance, the blank sample, which replaced the substrate with 50 μL of the buffer, was examined. Instead of test samples (acarbose), the positive control sample was composed of 10 L of DMSO (dimethyl sulfoxide). Assuming that A represents the absorbance of the control without test samples and B represents the absorbance with test samples present, the percentage of enzyme inhibition was computed as (1 B/A) 100. The analyses were completed in triplicate, and the mean SD of the outcomes was provided.

#### 4.3.2. Assay Protocol for Molecular Docking Study

To determine the binding affinities between newly accessible active scaffolds and the target α-glucosidase receptor active sites, docking investigations were carried out using the AutoDock Tools 1.5.6. on the Discovery Studio R2 64-bit Client system; interactive molecular structure visualization and analysis were performed. The RCSB Protein Data Bank (PDB) provided the α-glucosidase three-dimensional (3D) structure. The chemical structures of the ligands were obtained from the Pub Chem compound database. Using Open Babel, the PDB files were converted into PDBQT files. For docking analysis, the ligand and target PDB coordinates were optimized. The geometry was optimized using Gaussian 09 with the addition of the missing residues. These coordinates had the lowest energy and were the most stable. Further details were given in previously reported work [35,36].

## 5. Conclusions

In summary, a new class of functionalized imidazopyridine-bearing thiazole analogs was synthesized, and its α-glucosidase potency was exhibited. The spectral analysis, including ^1^H-NMR, ^13^C-NMR, and HREI-MS, confirmed the structures of the newly synthesized compounds. The in vitro α-glucosidase study against acarbose revealed that four newly synthesized analogs, **4a**, **4g**, **4o**, and **4p**, (bearing substituents either capable of forming hydrogen bonds or having strong EW natures) showed α-glucosidase activity with minimum IC_50_ values. Additionally, molecular docking studies revealed that these active analogs occupied the protein-binding pockets of α-glucosidase with strong binding interactions.

## Figures and Tables

**Table 1:** Different substituent(s) and MIC (α-glucosidase) values of synthesized imidazopyridine incorporating thiazole scaffolds (**4a-p**).

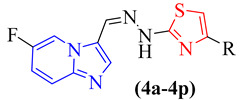					
S.NO	R	IC_50_ ± SEM ^a^ [μM]	S.NO	R	IC_50_ ± SEM ^a^ [μM]
**4a**	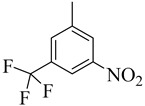	6.85 ± 2.18	**4i**	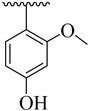	34.91 ± 5.84
**4b**	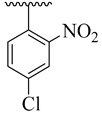	13.63 ± 1.67	**4j**	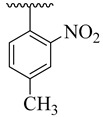	22.57 ± 3.55
**4c**	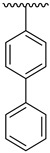	63.46 ± 5.28	**4k**	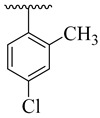	34.73 ± 5.23
**4d**	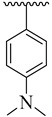	32.12 ± 4.29	**4l**	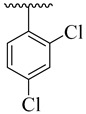	19.26 ± 2.58
**4e**	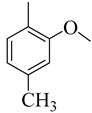	44.89 ± 5.27	**4m**	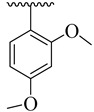	56.30 ± 5.94
**4f**	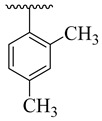	50.96 ± 5.80	**4n**	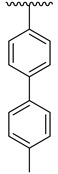	61.42 ± 4.56
**4g**	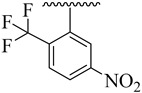	5.57 ± 3.45	**4o**	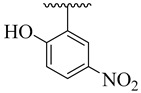	7.16 ± 1.40
**4h**	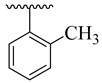	43.20 ± 6.16	**4p**	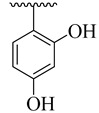	10.48 ± 2.20
^a^ Standard Acarbose drug					48.71 ± 2.65

**Table 2:** Representation of the types of interactions established by the most active analogs withthe active residue of the targeted enzyme from varied distances along with docking scores.

Active Analogs	Targeted Enzyme	Receptors	Types of Interactions	Distance (^o^A)	Docking Score
4g	α-glucosidase	GLU-A-595	Pi-Anion	6.52	−13.45
		GLU-A-595	Pi-Anion	6.85	
		PHE-A-200	Pi-Pi stacked	5.69	
		PHE-A-200	Pi-Pi stacked	4.80	
		VAL-A-96	H-B	3.98	
		VAL-A-96	Pi-R	5.34	
		PHE-A-95	Pi-R	6.69	
		PHE-A-95	Pi-R	4.18	
		PHE-A-95	H-F	4.87	
		HIS-A-94	Pi-R	5.48	
		HIS-A-94	H-F	6.00	
		HIS-A-94	H-F	4.66	
		PHE-A-95	C-H	3.93	
4a	α-glucosidase	ARG-A-91	Pi-R	5.47	−12.87
		TRP-A-90	Pi-S	7.20	
		TRP-A-90	Pi-R	6.58	
		PHE-A-200	H-F	4.04	
		PHE-A-200	Pi-R	5.41	
		VAL-A-201	Pi-R	4.94	
		VAL-A-96	Pi-R	4.88	
		HIS-A-94	H-F	5.80	
		HIS-A-94	Pi-R	5.84	
		GLN-A-202	H-B	4.09	
4o	α-glucosidase	GLU-A-595	Pi-anion	7.69	−12.15
		GLN-A-202	H-B	4.26	
		VAL-A-96	Pi-R	5.53	
		VAL-A-96	Pi-R	5.10	
		ARG-A-91	Pi-R	5.86	
		ARG-A-91	Pi-R	5.49	
		PHE-A-51	Pi-Pi T-shaped	5.47	
		HIS-A-94	C-H	6.18	
		HIS-A-94	Pi-Pi T-shaped	5.05	
4p	α-glucosidase	PHE-A-51	H-F	6.98	−11.25
		HIS-A-94	H-F	4.45	
		HIS-A-94	C-H	3.79	
		GLU-A-595	Pi-Anion	5.99	
		GLU-A-595	Pi-Anion	6.83	
		VAL-A-201	Pi-R	6.06	
		PHE-A-200	Pi-Pi T-shaped	5.99	
		VAL-A-96	Pi-R	6.24	
		ARG-A-91	Pi-R	5.93	

## Data Availability

Not applicable.

## Supplementary Materials

The following supporting information can be downloaded at: https://www.mdpi.com/article/10.3390/ph16091288/s1.
