# Supplementary material for: Imidazopyridine-Based Thiazole Derivatives as Potential Antidiabetic Agents: Synthesis, In Vitro Bioactivity, and In Silico Molecular Modeling Approach
Source: Pharmaceuticals (Basel). 2023 Sep 13;16(9):1288. doi: 10.3390/ph16091288 (PMC10535535; doi:10.3390/ph16091288)
Supplement: Supplementary file 1 [file pharmaceuticals-16-01288-s001.zip › pharmaceuticals-2568417-supplementary.pdf]

Imidazopyridine-Based Thiazole Derivatives as Potential Antidiabetic Agents: Synthesis, In Vitro Bioactivity, and In Silico Molecular Modeling Approach

4.3. Spectral Analysis

4.3.1. (Z)-2-(2-((6-fluoroimidazo[1,2-a]pyridin-3-yl)methylene)hydrazineyl)-4-(3-nitro-5-(trifluoromethyl)phenyl)thiazole (4a)

^{1}H-NMR (600 MHz, DMSO-d_6): δ 13.06 (s, 1H, N-H), 8.92 (s, 1H, Ar-H), 8.74 (s, 1H, Ar-H), 8.69 (s, 1H, Ar-H), 8.09 (s, 1H, H-C=N), 8.02 (s, 1H, imidazole-H), 7.93 (d, *J* = 7.0 Hz, 1H, imidazole-H), 7.85 (s, 1H, imidazole-H), 7.71 (s, 1H, thiazole-H), 7.52 (d, *J* = 7.8 Hz, 1H, imidazole-H); ^{13}C-NMR (150 MHz, DMSO-d_6): δ 182.1, 172.6, 169.3, 165.8, 164.4, 162.6, 159.9, 157.5, 153.0, 152.3, 148.7, 148.5, 138.7, 137.2, 136.4, 124.5, 124.3, 118.7; HREI-MS: m/z calcld for $C_{18}H_{10}F_4N_6SO_2$, $[M]^+$ 450.3795 Found 450.3758.

4.3.2. (Z)-4-(4-chloro-2-nitrophenyl)-2-(2-((6-fluoroimidazo[1,2-a]pyridin-3-yl)methylene)hydrazineyl)thiazole (4b)

^{1}H-NMR (600 MHz, DMSO-d_6): δ 13.11 (s, 1H, N-H), 8.87 (s, 1H, Ar-H), 8.52 (d, *J* = 8.3 Hz, 1H, Ar-H), 8.29 (s, 1H, H-C=N), 8.23 (d, *J* = 8.3 Hz, 1H, Ar-H), 8.05 (s, 1H, imidazole-H), 8.02 (d, *J* = 6.8 Hz, 1H, imidazole-H), 7.85 (s, 1H, imidazole-H), 7.83 (s, 1H, thiazole-H), 7.38 (d, *J* = 7.8 Hz, 1H, imidazole-H); ^{13}C-NMR (150 MHz, DMSO-d_6): δ 166.2, 163.1, 149.8, 141.2, 133.1, 131.5, 132.2, 131.6, 130.6, 127.6, 127.5, 122.4, 121.3, 120.9, 119.6, 111.0, 107.5; HREI-MS: m/z calcld for $C_{17}H_{10}FClN_6SO_2$, $[M]^+$ 416.8705 Found 416.8678.

4.3.3. (Z)-4-([1,1′-biphenyl]-4-yl)-2-(2-((6-fluoroimidazo[1,2-a]pyridin-3-yl)methylene)hydrazineyl)thiazole (4c)

^{1}H-NMR (600 MHz, DMSO-d_6): δ 13.34 (s, 1H, N-H), 8.92 (d, *J* = 7.4 Hz, 2H, Ar-H (A)), 8.89 (s, 1H, H-C=N), 8.75 (d, *J* = 7.3 Hz, 2H, Ar-H (A)), 8.69 (s, 1H, imidazole-H), 8.63 (d, *J* = 8.2 Hz, 2H, Ar-H (B)), 8.24 (d, *J* = 7.6 Hz, 1H, imidazole-H), 8.03 (d, *J* = 7.2 Hz, 2H, Ar-H (B)), 7.85 (s, 1H, imidazole-H), 7.73-7.82 (m, 1H, Ar-H (B)) 7.69 (s, 1H, thiazole-H), 7.32 (d, *J* = 7.2 Hz, 1H, imidazole-H); ^{13}C-NMR (150 MHz, DMSO-d_6): δ 179.8, 173.6, 168.3, 165.5, 164.2, 163.8, 160.7, 160.2, 158.7, 156.2, 153.8, 153.4, 149.8, 148.9, 143.4, 142.3, 141.6, 139.8, 137.6, 136.8, 131.3, 123.9, 121.3; HREI-MS: m/z calcld for $C_{23}H_{16}FN_5S$, $[M]^+$ 413.8752 Found 413.8723.

4.3.4. (Z)-4-(2-(2-((6-fluoroimidazo[1,2-a]pyridin-3-yl)methylene)hydrazineyl)thiazol-4-yl)-N,N-dimethylaniline (4d)

^{1}H-NMR (600 MHz, DMSO-d_6): δ 13.26 (s, 1H, N-H), 8.52 (s, 1H, H-C=N), 8.05 (s, 1H, imidazole-H), 7.86 (d, 2H, Ar-H), 7.80 (d, *J* = 7.0 Hz, 1H, imidazole-H), 7.68 (s, 1H, imidazole-H), 7.63 (s, 1H, thiazole-H), 7.57 (d, *J* = 8.2 Hz, 2H, Ar-H), 7.38 (d, *J* = 7.8 Hz, 1H, imidazole-H), 3.60 (s, 6H, -N(CH_3)$_2$);^{13}C-NMR (150 MHz, DMSO-d_6): δ 186.4, 176.8, 173.7, 163.9, 163.4, 160.6, 158.2, 155.7, 154.6, 150.4, 150.2, 142.5, 156.6, 147.7, 134.5, 133.2, 120.8, 58.5, 58.3; HREI-MS: m/z calcld for $C_{19}H_{17}FN_6S$, [M]$^+$ 380.4562 Found 380.4538.

4.3.5. (Z)-2-(2-((6-fluoroimidazo[1,2-a]pyridin-3-yl)methylene)hydrazineyl)-4-(2-methoxy-4-methylphenyl)thiazole (4e)

^{1}H-NMR (600 MHz, DMSO-d_6): δ 13.10 (s, 1H, N-H), 8.79 (s, 1H, H-C=N), 8.24 (s, 1H, imidazole-H), 8.05 (d, *J* = 8.6 Hz, 1H, Ar-H), 7.93 (d, *J* = 7.0 Hz, 1H, imidazole-H), 7.90 (s, 1H, imidazole-H), 7.83 (s, 1H, thiazole-H), 7.81 (s, 1H, Ar-H), 8.60 (d, *J* = 8.3 Hz, 1H, Ar-H), 7.54 (d, *J* = 7.8 Hz, 1H, imidazole-H), 4.19 (s, 3H, Ar-OCH_3), 2.95 (s, 3H, Ar-CH_3); ^{13}C-NMR (150 MHz, DMSO-d_6): δ 188.4, 180.5, 173.6, 171.5, 168.3, 164.6, 163.4, 156.5, 156.0, 153.3, 151.7,150.7, 146.2, 143.4, 134.2, 129.8, 115.0, 63.4, 31.7; HREI-MS: m/z calcld for $C_{19}H_{16}FN_5SO$, [M]$^+$ 381.8345 Found 381.8317.

4.3.6. (Z)-4-(2,4-dimethylphenyl)-2-(2-((6-fluoroimidazo[1,2-a]pyridin-3-yl)methylene)hydrazineyl)thiazole (4f)

^{1}H-NMR (600 MHz, DMSO-d_6): δ 13.14 (s, 1H, N-H), 8.67 (s, 1H, H-C=N), 8.29 (s, 1H, imidazole-H), 7.97 (d, *J* = 6.9 Hz, 1H, imidazole-H), 8.35 (d, *J* = 7.8 Hz, 1H, Ar-H), 7.84 (s, 1H, imidazole-H), 8.68 (s, 1H, Ar-H), 8.60 (d, *J* = 8.3 Hz, 1H, Ar-H), 7.71 (s, 1H, thiazole-H), 7.54 (d, *J* = 7.8 Hz, 1H, imidazole-H), 2.94 (s, 3H, Ar-CH_3), 2.82 (s, 3H, Ar-CH_3); ^{13}C-NMR (150 MHz, DMSO-d_6): δ 165.9, 163.1, 151.1, 141.2, 137.2, 131.9, 131.2, 130.4, 129.3, 129.2, 125.6, 125.4, 122.3, 119.8, 119.7, 111.0, 105.8, 30.6, 20.7; HREI-MS: m/z calcld for $C_{19}H_{16}FN_5S$, [M]$^+$ 365.7398 Found 365.7369.

4.3.7. (Z)-2-(2-((6-fluoroimidazo[1,2-a]pyridin-3-yl)methylene)hydrazineyl)-4-(5-nitro-2-(trifluoromethyl)phenyl)thiazole (4g)

^{1}H-NMR (600 MHz, DMSO-d_6): δ 13.20 (s, 1H, N-H), 8.67 (s, 1H, Ar-H), 8.35 (d, *J* = 7.8 Hz, 1H, Ar-H), 8.67 (s, 1H, H-C=N), 8.60 (d, *J* = 8.3 Hz, 1H, Ar-H), 8.29 (s, 1H, imidazole-H), 7.97 (d, *J* = 6.9 Hz, 1H, imidazole-H), 7.84 (s, 1H, imidazole-H), 7.71 (s, 1H, thiazole-H), 7.54 (d, *J* = 7.8 Hz, 1H,imidazole-H); ^{13}C-NMR (150 MHz, DMSO-d_6): δ 181.8, 170.2, 167.5, 163.7, 162.6, 162.2, 157.2, 156.9, 150.9, 143.5, 140.5, 140.2, 133.7, 131.2, 129.2, 128.8, 121.3, 113.5; HREI-MS: m/z calcld for $C_{18}H_{10}F_4N_6SO_2$, [M]$^+$450.3365 Found 450.3341.

4.3.8. (Z)-2-(2-((6-fluoroimidazo[1,2-a]pyridin-3-yl)methylene)hydrazineyl)-4-(o-tolyl)thiazole (4h)

^{1}H-NMR (600 MHz, DMSO-d_6): δ 13.30 (s, 1H, N-H), 8.62 (s, 1H, H-C=N), 8.29 (s, 1H, imidazole-H), 8.21 (dd, *J* = 7.5 Hz, 1H, Ar-H), 8.07 (d, *J* = 7.0 Hz, 1H, imidazole-H), 7.84 (s, 1H, imidazole-H), 7.65-7.72 (m, 1H, Ar-H), 7.63 (dd, *J* = 6.9 Hz, 1H, Ar-H), 7.52-7.60 (m, 1H, Ar-H), 7.41 (s, 1H, thiazole-H), 7.33 (d, *J* = 7.8 Hz, 1H, imidazole-H), 3.19 (s, 3H, Ar-CH_3); ^{13}C-NMR (150 MHz, DMSO-d_6): δ 179.6, 174.8, 171.5, 168.5, 166.8, 164.3, 161.4, 159.8, 158.0, 157.5, 152.6, 144.6, 139.6, 138.8, 128.5, 119.4, 109.7, 28.7; HREI-MS: m/z calcld for $C_{18}H_{14}FN_5S$, $[M]^+$ 351.2542 Found 351.2510.

4.3.9. *(Z)-4-(2-(2-((6-fluoroimidazo[1,2-a]pyridin-3-yl)methylene)hydrazineyl)thiazol-4-yl)-3-methoxyphenol (4i)*

^{1}H-NMR (600 MHz, DMSO-d_6): δ 13.34 (s, 1H, N-H), 11.25 (s, 1H, Ar-OH), 7.79 (s, 1H, H-C=N), 7.72 (s, 1H, imidazole-H), 7.54 (d, *J* = 7.3 Hz, 1H, Ar-H), 7.36 (s, 1H, imidazole-H), 7.29 (s, 1H, thiazole-H), 7.27 (d, *J* = 6.9 Hz, 1H, imidazole-H), 7.24 (d, *J* = 7.3 Hz, 1H, imidazole-H), 7.11 (s, 1H, Ar-H), 6.96 (d, *J* = 8.3 Hz, 1H, Ar-H), 2.50 (s, 3H, Ar-OCH_3); ^{13}C-NMR (150 MHz, DMSO-d_6): δ 177.4, 175.3, 167.7, 166.9, 160.5, 157.4, 156.7, 149.5, 143.6, 140.3, 140.1, 134.8, 131.5, 130.9, 129.5, 125.3, 113.1, 63.7; HREI-MS: m/z calcld for $C_{18}H_{14}FN_5SO_2$, $[M]^+$ 383.3365 Found 383.3341.

4.3.10. *(Z)-2-(2-((6-fluoroimidazo[1,2-a]pyridin-3-yl)methylene)hydrazineyl)-4-(4-methyl-2-nitrophenyl)thiazole (4j)*

^{1}H-NMR (600 MHz, DMSO-d_6): δ 13.33 (s, 1H, N-H), 8.32 (d, *J* = 7.7 Hz, 1H, Ar-H), 8.02 (s, 1H, H-C=N), 8.23 (s, 1H, imidazole-H), 8.05 (s, 1H, Ar-H), 7.99 (d, *J* = 5.9 Hz, 1H, imidazole-H), 7.87 (d, *J* = 8.3 Hz, 1H, Ar-H), 7.84 (s, 1H, imidazole-H), 7.51 (s, 1H, thiazole-H), 7.47 (d, *J* = 7.3 Hz, 1H, imidazole-H), 2.84 (s, 3H, Ar-CH_3); ^{13}C-NMR (150 MHz, DMSO-d_6): δ 185.9, 178.6, 173.2, 171.5, 169.4, 168.6, 168.3, 165.5, 158.8, 157.7, 147.4, 145.6, 141.7, 139.5, 132.5, 130.3, 128.1, 35.2; HREI-MS: m/z calcld for $C_{18}H_{13}FN_6SO_2$, $[M]^+$ 396.1353 Found 396.1322.

4.3.11. *(Z)-4-(4-chloro-2-methylphenyl)-2-(2-((6-fluoroimidazo[1,2-a]pyridin-3-yl)methylene)hydrazineyl)thiazole (4k)*

^{1}H-NMR (600 MHz, DMSO-d_6): δ 13.19 (s, 1H, N-H), 8.27 (s, 1H, H-C=N), 8.23 (s, 1H, imidazole-H), 8.19 (d, *J* = 7.7 Hz, 1H, Ar-H), 7.93 (d, *J* = 7.0 Hz, 1H, imidazole-H), 7.89 (s, 1H, Ar-H), 7.84 (s, 1H, imidazole-H), 7.72 (s, 1H, thiazole-H), 7.68 (d, *J* = 7.1 Hz, 1H, imidazole-H), 7.41 (d, *J* = 7.9 Hz, 1H, Ar-H), 2.84 (s, 3H, Ar-CH_3); ^{13}C-NMR (150 MHz, DMSO-d_6): δ 187.2, 178.3, 173.8, 171.5, 168.9, 165.2, 160.3, 159.9, 157.5, 157.1, 156.5, 147.3, 142.2, 139.3, 130.4, 129.3, 123.7, 32.8; HREI-MS: m/z calcld for $C_{18}H_{13}FClN_5S$, $[M]^+$ 385.5309 Found 385.5288.

4.3.12. *(Z)-4-(2,4-dichlorophenyl)-2-(2-((6-fluoroimidazo[1,2-a]pyridin-3-yl)methylene)hydrazineyl)thiazole (4l)*

^{1}H-NMR (600 MHz, DMSO-d_6): δ 13.25 (s, 1H, N-H), 8.30 (d, J = 6.9 Hz, 1H, Ar-H), 8.09 (s, 1H, H-C=N), 8.07 (s, 1H, imidazole-H), 7.99 (d, J = 6.8 Hz, 1H, imidazole-H), 7.82 (s, 1H, Ar-H), 7.71 (s, 1H, imidazole-H), 7.68 (s, 1H, thiazole-H), 7.53 (d, J = 7.1 Hz, 1H, imidazole-H), 7.36 (d, J = 7.1 Hz, 1H, Ar-H); ^{13}C-NMR (150 MHz, DMSO-d_6): δ 176.8, 174.3, 173.7, 169.3, 168.8, 166.9, 162.2, 159.1, 156.3, 154.7, 151.7, 141.5, 140.6, 140.3, 135.5, 131.3, 126.7; HREI-MS: m/z calcld for $C_{17}H_{10}FCl_2N_5S$, $[M]^+$ 406.3493 Found 406.3462.

4.3.13. (Z)-4-(2,4-dimethoxyphenyl)-2-(2-((6-fluoroimidazo[1,2-a]pyridin-3-yl)methylene)hydrazineyl)thiazole (4m)

^{1}H-NMR (600 MHz, DMSO-d_6): δ 13.16 (s, 1H, N-H), 8.21 (s, 1H, H-C=N), 8.13 (s, 1H, imidazole-H), 8.09 (d, J = 6.9 Hz, 1H, Ar-H), 7.92 (d, J = 8.0 Hz, 1H, imidazole-H), 7.68 (s, 1H, imidazole-H), 7.54 (s, 1H, thiazole-H), 7.10 (d, J = 6.4 Hz, 1H, imidazole-H), 6.87 (d, J = 7.1 Hz, 1H, Ar-H), 6.82 (s, 1H, Ar-H), 4.02 (s, 3H, Ar-OCH_3), 3.97 (s, 3H, Ar-OCH_3); ^{13}C-NMR (150 MHz, DMSO-d_6): δ 163.1, 153.0, 150.9, 146.7, 141.2, 131.8, 130.4, 122.8, 122.3, 119.7, 114.0, 112.8, 111.0, 111.0, 55.8, 55.4; HREI-MS: m/z calcld for $C_{19}H_{16}FN_5O_2S$, $[M]^+$ 397.6189 Found 397.6142.

4.3.14. (Z)-2-(2-((6-fluoroimidazo[1,2-a]pyridin-3-yl)methylene)hydrazineyl)-4-(4′-methyl-[1,1′-biphenyl]-4-yl)thiazole (4n)

^{1}H-NMR (600 MHz, DMSO-d_6): δ 13.29 (s, 1H, N-H), 8.86 (d, J = 7.0 Hz, 2H, Ar-H (A)), 8.82 (s, 1H, H-C=N), 8.45 (d, J = 7.0 Hz, 2H, Ar-H (A)), 8.38 (s, 1H, imidazole-H), 8.30 (d, J = 7.4 Hz, 1H, imidazole-H), 7.82 (s, 1H, imidazole-H), 8.13 (d, J = 8.0 Hz, 2H, Ar-H (B)), 8.00 (d, J = 7.2 Hz, 2H, Ar-H (B)), 7.93 (s, 1H, thiazole-H), 7.58 (d, J = 7.2 Hz, 1H, imidazole-H), 3.12 (s, 3H, Ar-CH_3 (B));^{13}C-NMR (150 MHz, DMSO-d_6): δ 173.8, 167.6, 167.3, 163.7, 162.3, 162.8, 160.7, 157.6, 155.8, 154.4, 151.3, 149.6, 147.8, 144.2, 142.6, 142.3, 139.7, 137.9, 135.4, 135.2, 131.2, 128.5, 116.7, 34.3; HREI-MS: m/z calcld for $C_{24}H_{18}FN_5S$, $[M]^+$ 427.8752 Found 427.8723.

4.3.15. (Z)-2-(2-(2-((6-fluoroimidazo[1,2-a]pyridin-3-yl)methylene)hydrazineyl)thiazol-4-yl)-4-nitrophenol (4o)

^{1}H-NMR (600 MHz, DMSO-d_6): δ 13.34 (s, 1H, N-H), 11.25 (s, 1H, Ar-OH), 7.86 (s, 1H, Ar-H), 7.69 (d, J = 7.3 Hz, 2H, imidazole-H), 7.66 (s, 1H, H-C=N), 7.60 (s, 1H, imidazole-H), 7.54 (d, J = 7.3 Hz, 1H, Ar-H), 7.35 (s, 1H, imidazole-H), 7.10 (s, 1H, thiazole-H), 6.96 (d, J = 7.8 Hz, 1H, Ar-H); ^{13}C-NMR (150 MHz, DMSO-d_6): δ 184.9, 179.0, 173.5, 172.8, 170.3, 166.0, 162.6, 160.9, 156.2, 155.3, 151.7, 143.9, 140.5, 139.3, 136.6, 132.8, 128.2; HREI-MS: m/z calcld for $C_{17}H_{11}FN_6O_3S$, $[M]^+$ 398.1475 Found 398.1445.

4.3.16. (Z)-4-(2-(2-((6-fluoroimidazo[1,2-a]pyridin-3-yl)methylene)hydrazineyl)thiazol-4-yl)benzene-1,3-diol (4p)

^{1}H-NMR (600 MHz, DMSO-d_6): δ 13.24 (s, 1H, N-H), 11.67 (s, 1H, Ar-OH), 10.03 (s, 1H, Ar-OH), 8.14 (s, 1H, H-C=N), 8.02 (s, 1H, imidazole-H), 7.86 (d, J = 8.2 Hz, 1H, imidazole-H), 7.53 (s, 1H, imidazole-H), 7.49 (d,

J = 7.0 Hz, 1H, Ar-H), 7.17 (s, 1H, thiazole-H), 7.06 (d, *J* = 7.2 Hz, 1H, imidazole-H), 6.89 (d, *J* = 7.0 Hz, 1H, Ar-H), 7.17 (s, 1H, Ar-H); ^{13}C-NMR (150 MHz, DMSO-d_6): δ 183.2, 175.7, 174.6, 174.0, 172.3, 165.5, 161.5, 160.3, 153.1, 150.3, 148.5, 146.3, 145.5, 137.3, 133.7, 130.8, 123.8; HREI-MS: m/z calcld for $C_{17}H_{12}FN_5O_2S$, $[M]^+$ 369.0164 Found 369.0130.

4.4. *Assay protocol for α-glucosidase inhibition*

The α-glucosidase inhibition profiles of synthesized scaffolds were determined spectrophotometrically, with some modifications to an earlier method [1]. For each chemical, 10 µL of the sample, 120 µL of 100 mM potassium phosphate buffer, and 20 µL of 0.5 U/mL alpha-glucosidase solution was mixed (0.3 mM, in buffer). Acarbose was frequently used as an inhibitor. At 37 °C, each mixture was incubated for a separate 15 minutes. The enzymatic reaction was initiated by adding 20 µL of the substrate 4-nitrophenyl—D-α-glucopyranoside (pNPG) (5 mM) to the solution. The mixture was once more incubated at 37 °C for 15 minutes. To stop the process at that point, 80µL of sodium carbonate solution were injected (0.2 M). Additionally, the mixture without any enzymes served as a blank sample. The absorbance was finally determined at 405 nm. The blank sample, in which the substrate was swapped out for 50 µL of the buffer, was analyzed to properly quantify the background absorbance. The positive control sample was made up of 10 L of DMSO (dimethyl sulfoxide) rather than test samples (acarbose). The percentage of enzyme inhibition was calculated as (1 B/A) 100, assuming that A represents the absorbance of the control without test samples and B represents the absorbance with test samples present. The analyses were carried out in triplicate, and the results were presented as mean SD.

4.5. *Assay protocol for molecular docking study*

Using the AutoDock Tools 1.5.6 Workspace, docking tests were performed to determine the binding affinities between newly afforded active scaffolds and target α-glucosidase receptor actives sites [84]. On the Discovery Studio R2 64-bit Client system, interactive molecular structure visualization and analysis were done. From the RCSB Protein Data Bank, the three-dimensional (3D) structure of α-glucosidase was retrieved (PDB). The chemical structures of the ligands were obtained from the Pub Chem compound database. Using Open Babel, the PDB files were converted into PDBQT files. For docking analysis, the PDB coordinates of the ligands and targets were optimized. Geometry optimization is carried out using Gaussian 09 (adding missing residues). These coordinates had the least energy and the most stable conformation. Further details were summarized in our previous articles [2,3].

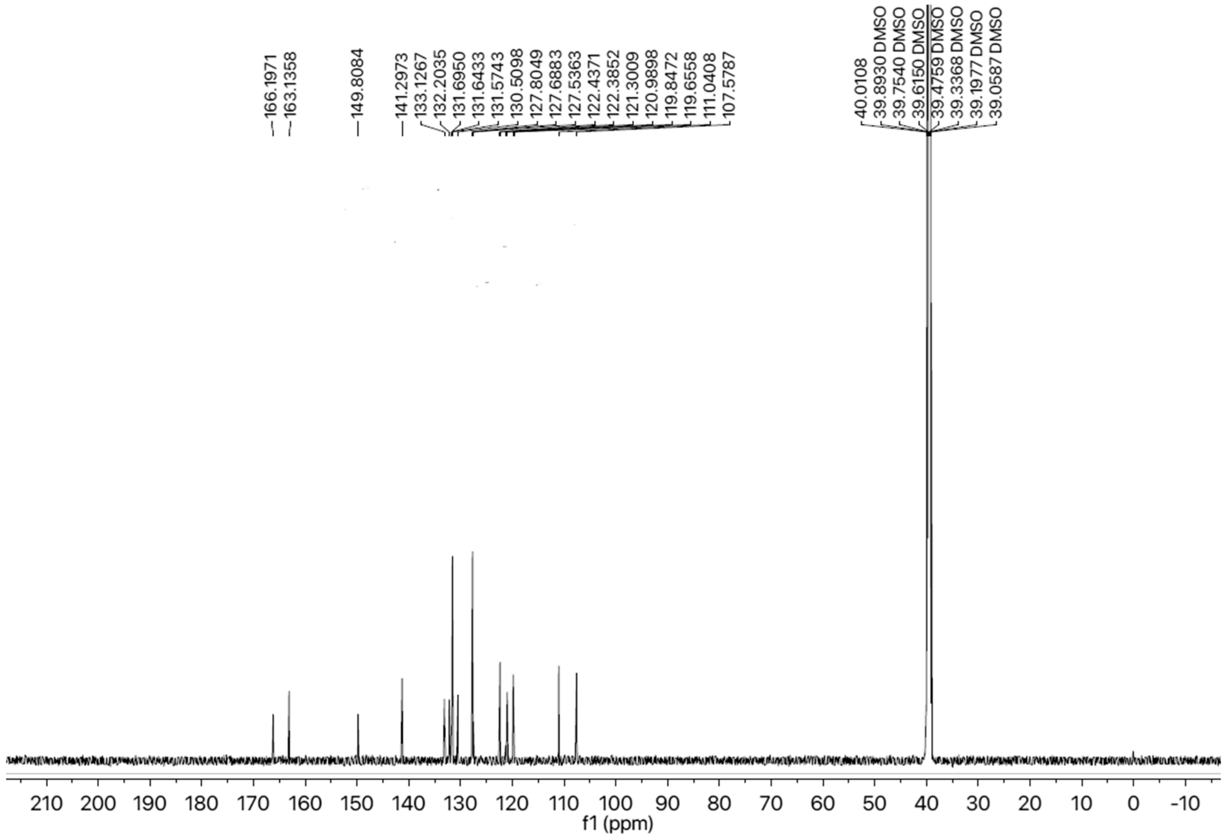

Figure S1. ^{13}CNMR for the compound **4b** *(Z)-4-(4-chloro-2-nitrophenyl)-2-(2-((6-fluoroimidazo[1,2-a]pyridin-3-yl)methylene)hydrazineyl)thiazole (4b)*

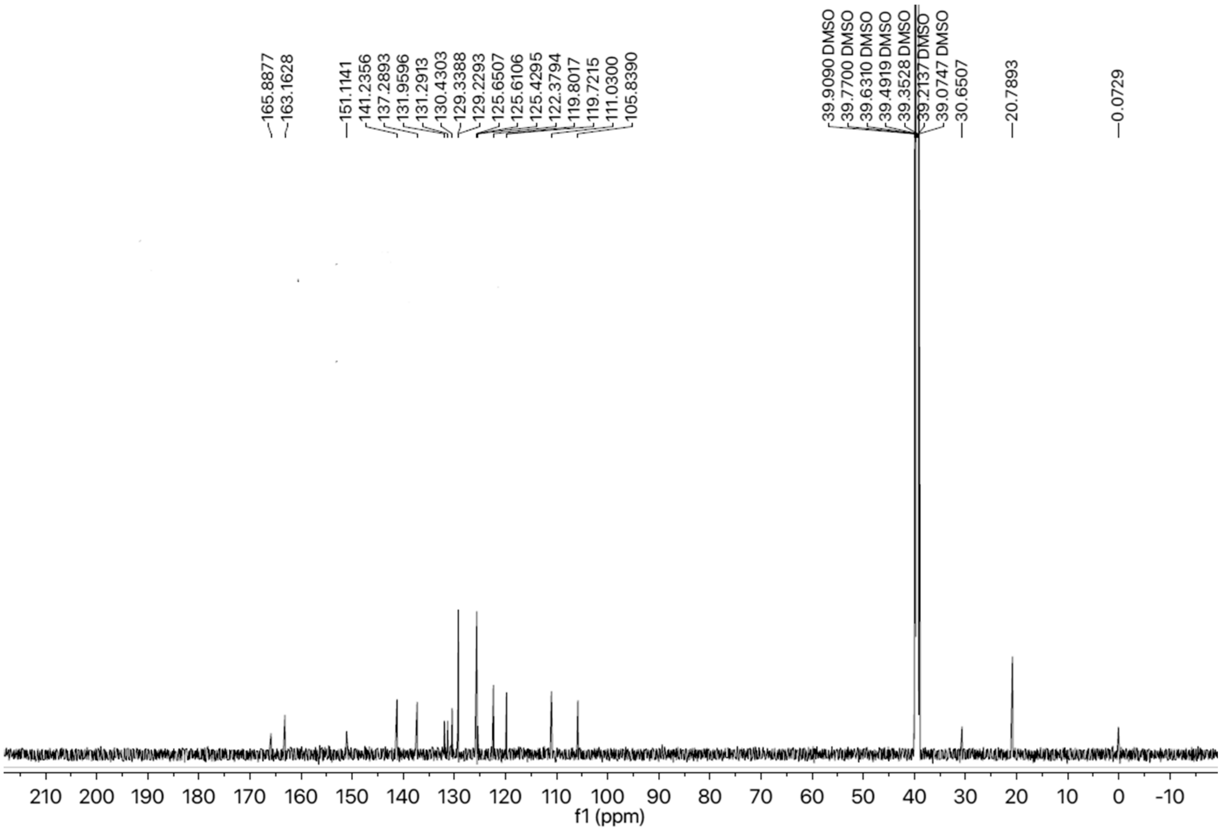

Figure S2. ^{13}CNMR for the compound **4f** *(Z)-4-(2,4-dimethylphenyl)-2-(2-((6-fluoroimidazo[1,2-a]pyridin-3-yl)methylene)hydrazineyl)thiazole*

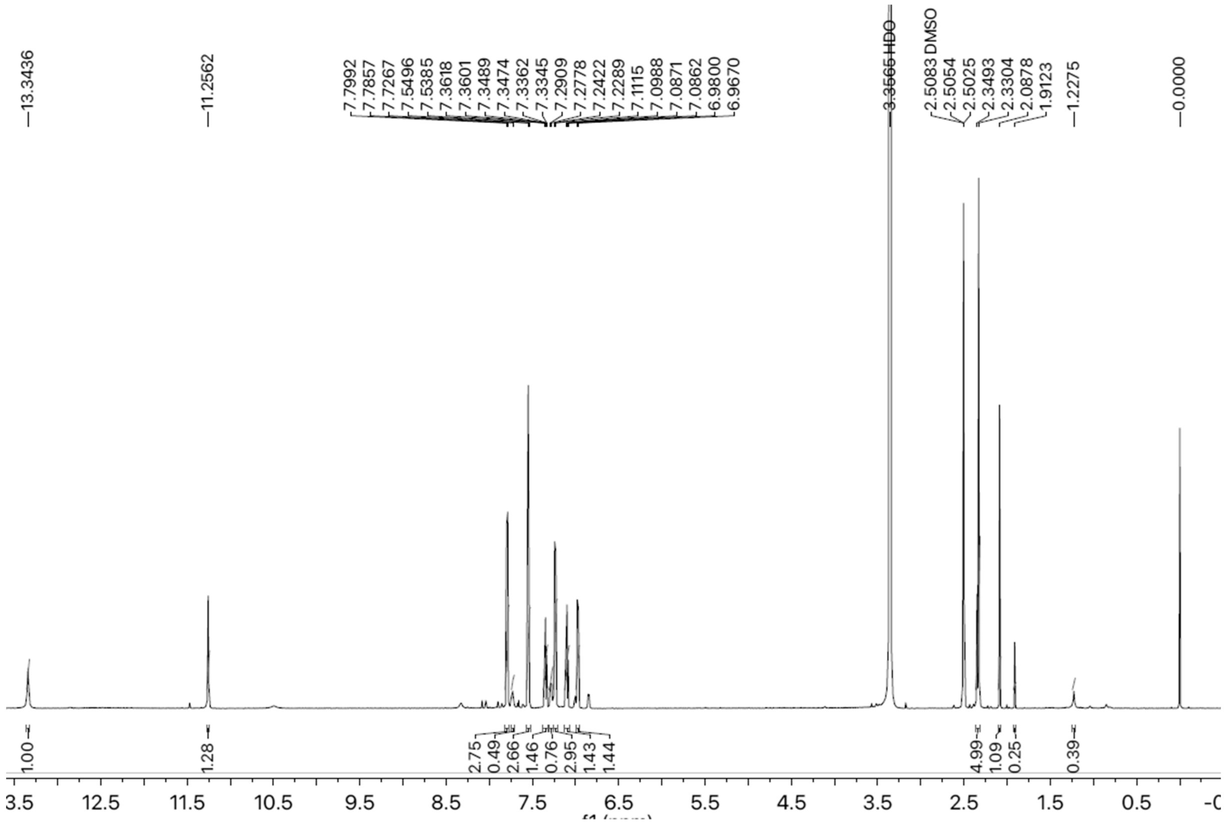

Figure S3. ^{1}HNMR for the compound **4i** *(Z)-4-(2-(2-((6-fluoroimidazo[1,2-a]pyridin-3-yl)methylene)hydrazineyl)thiazol-4-yl)-3-methoxyphenol (4i)*

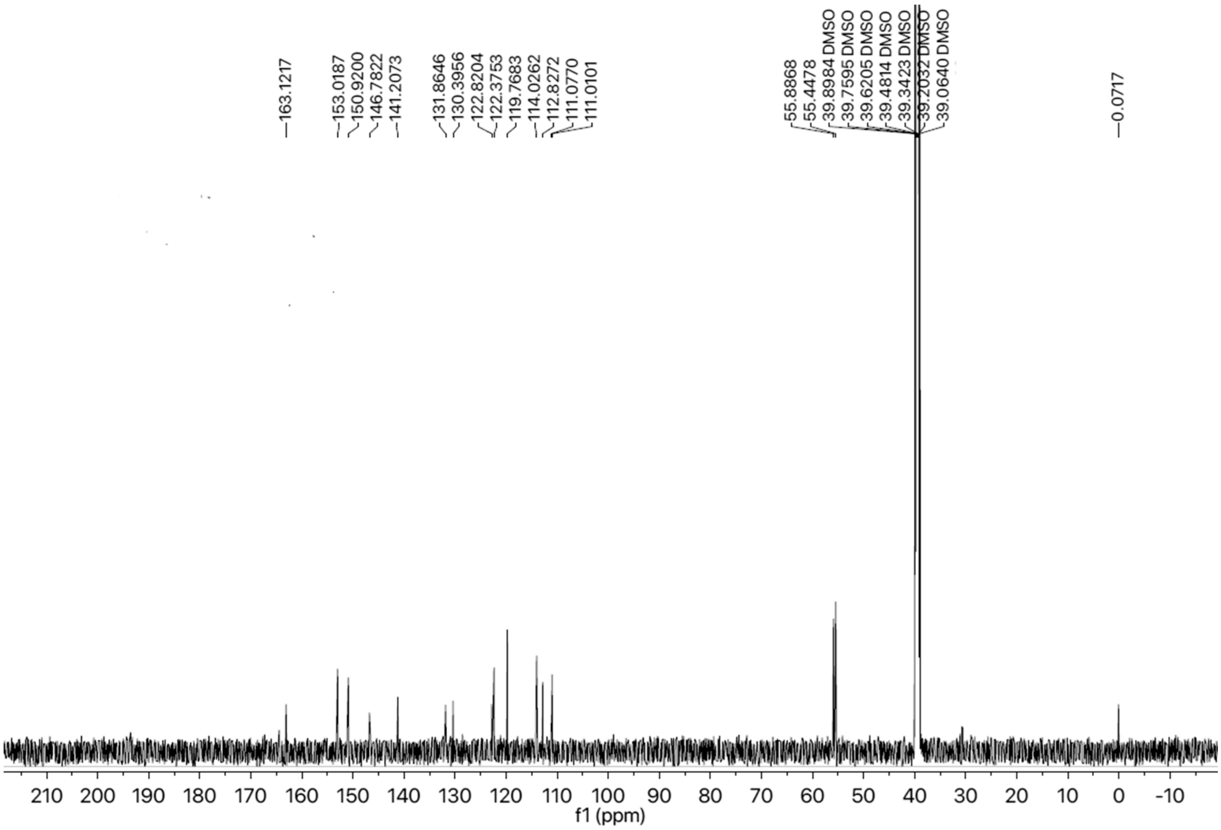

Figure S4. ^{13}CNMR for the compound **4m** *(Z)-4-(2,4-dimethoxyphenyl)-2-(2-((6-fluoroimidazo[1,2-a]pyridin-3-yl)methylene)hydrazineyl)thiazole (4m)*

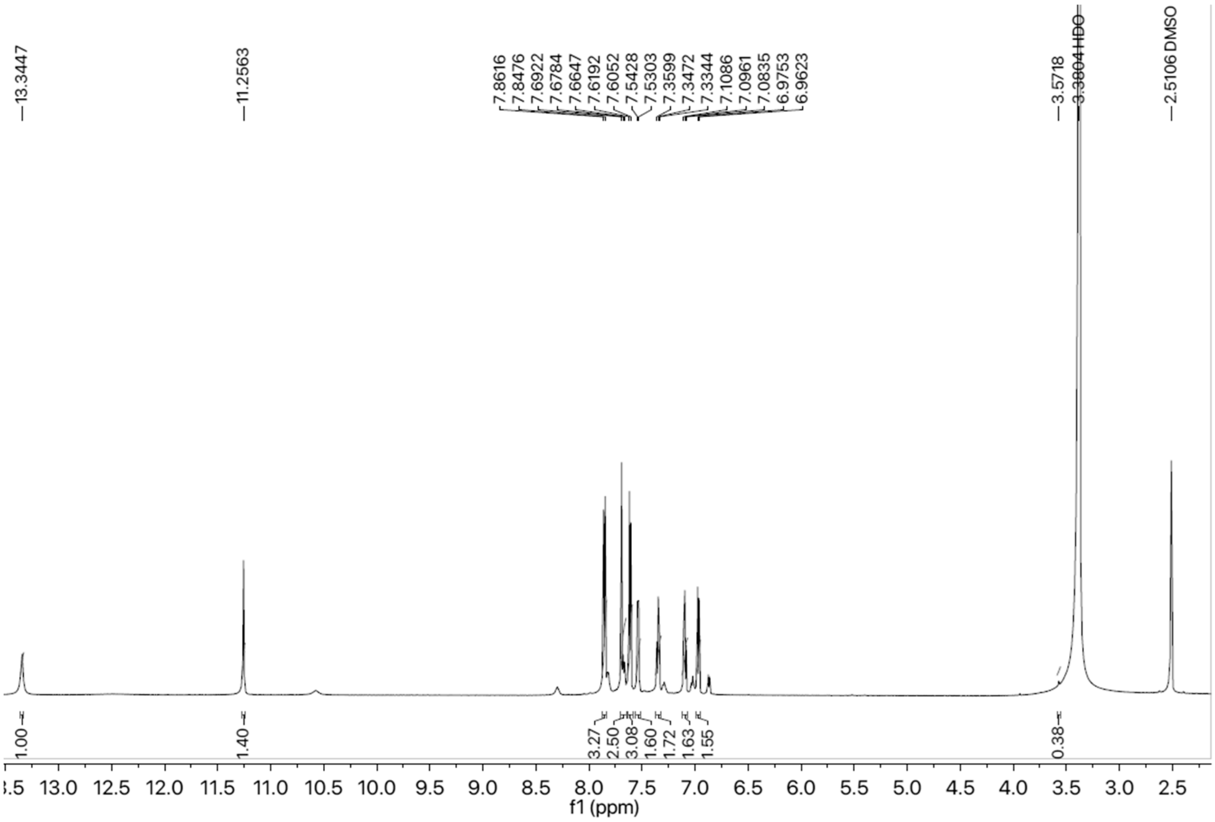

Figure S5. ^{1}HNMR for the compound **4o** *(Z)-2-(2-(2-((6-fluoroimidazo[1,2-a]pyridin-3-yl)methylene)hydrazineyl)thiazol-4-yl)-4-nitrophenol (4o)*

References

1. Eskandani, M.; Babak Bahadori, M.; Zengin, G.; Dinparast, L.; Bahadori, S. Novel natural agents from Lamiaceae family: An evaluation on toxicity and enzyme inhibitory potential linked to diabetes mellitus. *Current Bioactive Compounds* **2016**, *12*, 34-38.
2. Khan, S.; Iqbal, S.; Rahim, F.; Shah, M.; Hussain, R.; Alrbyawi, H.; Rehman, W.; Dera, A.A.; Rasheed, L.; Somaily, H. New Biologically Hybrid Pharmacophore Thiazolidinone-Based Indole Derivatives: Synthesis, In Vitro Alpha-Amylase and Alpha-Glucosidase along with Molecular Docking Investigations. *Molecules* **2022**, *27*, 6564.
3. Khan, S.; Iqbal, S.; Shah, M.; Rehman, W.; Hussain, R.; Rasheed, L.; Alrbyawi, H.; Dera, A.A.; Alahmdi, M.I.; Pashameah, R.A. Synthesis, In Vitro Anti-Microbial Analysis and Molecular Docking Study of Aliphatic Hydrazide-Based Benzene Sulphonamide Derivatives as Potent Inhibitors of α-Glucosidase and Urease. *Molecules* **2022**, *27*, 7129.
